# Time‐course analysis of cerebral circulation and cardiorespiratory responses to acute central blood volume reduction in healthy young males

**DOI:** 10.1113/EP092693

**Published:** 2025-10-10

**Authors:** Marina Feeley, Tomoki Watada, Go Ito, Ai Shimada, Toru Sawai, Hideomi Nakata, Shingo Otsuki, Tadayoshi Miyamoto

**Affiliations:** ^1^ Graduate School of Human Environment Osaka Sangyo University Osaka Japan; ^2^ Faculty of Sport and Health Sciences Osaka Sangyo University Osaka Japan

**Keywords:** blood pressure, cerebral blood velocity, lower‐body negative pressure, orthostatic hypotension, respiration

## Abstract

Central blood volume (CBV) reduction challenges circulatory and respiratory homeostasis, particularly during the initial compensatory phase (0–2 min), when rapid physiological adaptations occur. In this study, we examined dynamic cardiorespiratory responses to CBV reduction using lower‐body negative pressure (LBNP) in 11 healthy young males. Participants completed three standardized 2 min LBNP trials at −45 mmHg, with respiratory variables assessed via flow measurement and breath‐by‐breath gas analysis, while cardiovascular parameters and cerebral blood flow were monitored using ECG, blood pressure and transcranial Doppler ultrasonography. During LBNP exposure, thoracic admittance, an indicator of CBV reduction, decreased by 13.4% (*p* < 0.001), indicating significant CBV reduction. Following rigorous statistical correction for multiple comparisons, time‐course analysis revealed that mean blood pressure decreased temporarily during the initial phase (0–30 s), whereas heart rate increased progressively (16.4%, *p* < 0.001). End‐tidal $P_{CO_{2}}$ showed a consistent reduction (5.9%, *p* < 0.001), whereas minute ventilation and middle cerebral artery mean blood velocity showed no significant changes after statistical correction (−9.3% and −5.0%, respectively, *p* > 0.05). Exploratory correlation analysis revealed a significant negative correlation between mean blood pressure and tidal volume during the initial phase only (*r* = −0.78, *p* = 0.004). Cross‐correlation analysis suggested temporal patterns between respiratory and cerebrovascular responses, with respiratory changes preceding cerebrovascular adjustments by 10–20 s. These findings, along with individual variability, suggest rapid cardiorespiratory and cerebrovascular interactions during orthostatic stress, demonstrating dynamic cardiovascular and respiratory responses with distinct temporal patterns that provide insights into physiological mechanisms maintaining homeostasis during gravitational stress.

## Abstract (highlights)

- **What is the central question of this study?**
  How do integrated cardiorespiratory and cerebrovascular systems respond during the crucial first 2 min of acute central blood volume reduction in healthy young males?
- **What is the main finding and its importance?**
  We identified distinct temporal compensatory mechanisms: a strong negative correlation between mean blood pressure and tidal volume (*r* = −0.78) during the initial 30 s, with respiratory changes preceding cerebrovascular responses by 10–20 s, providing new insights into rapid physiological adaptations to orthostatic stress.

## 1 INTRODUCTION

Central blood volume (CBV) reduction challenges circulatory and respiratory systems, particularly during gravitational stress, spaceflight and orthostatic hypotension. Lower‐body negative pressure (LBNP) has been widely used to simulate these conditions. Although many studies have investigated the steady‐state physiological effects of LBNP, the immediate, dynamic responses that occur during the first moments of CBV reduction remain poorly characterized. This initial period is crucial because compensatory mechanisms are rapidly recruited to maintain cardiovascular and cerebral homeostasis.

The physiological cascade initiated by CBV reduction involves complex, interconnected responses across multiple systems. When CBV decreases during orthostatic stress, the immediate haemodynamic consequence is reduced venous return and cardiac output, triggering baroreceptor‐mediated cardiovascular adjustments including increased heart rate and peripheral vasoconstriction. These haemodynamic adjustments also influence respiratory control (Goswami et al., 2019). Animal studies have demonstrated that carotid baroreceptor activation can directly affect breathing patterns (Brunner et al., 1982), and CBV reduction affects ventilation–perfusion matching in the lungs, potentially altering gas exchange efficiency.

One consistent finding in studies involving CBV reduction is a decline in the partial pressure of end‐tidal CO_2_ ($P_{\mathrm{ETC}O_{2}}$), despite little or no change in minute ventilation (${\dot{V}}_{E}$). CBV reduction consistently decreases $P_{\mathrm{ETC}O_{2}}$, as demonstrated across multiple experimental protocols, including LBNP and water‐immersion studies (Feeley et al., 2024; Ogoh et al., 2013., Miyamoto et al., 2014). This reduction in $P_{\mathrm{ETC}O_{2}}$ appears to result from altered pulmonary perfusion patterns and modified respiratory chemoreflex sensitivity rather than changes in ${\dot{V}}_{E}$ per se. The decrease in $P_{\mathrm{ETC}O_{2}}$ during CBV reduction has important implications for cerebral blood flow regulation, because CO_2_ is a potent cerebrovascular dilator that rapidly modulates cerebral perfusion to maintain arterial pH homeostasis (Aaslid et al., 1989). The relationship between CBV reduction, $P_{\mathrm{ETC}O_{2}}$ changes and cerebral blood flow presents a physiological paradox. Although reduced $P_{\mathrm{ETC}O_{2}}$ would typically cause cerebral vasoconstriction and decreased cerebral blood flow, orthostatic stress simultaneously activates compensatory mechanisms that might preserve cerebral perfusion. Previous studies have shown that changes in ${\dot{V}}_{E}$ during LBNP are more strongly correlated with middle cerebral artery mean blood velocity (MCA *V*_mean_) than with $P_{\mathrm{ETC}O_{2}}$ changes, suggesting that cerebral CO_2_ clearance might be compromised during CBV reduction, potentially leading to compensatory hyperventilation (Ogoh et al., 2013). Furthermore, slow breathing techniques can enhance orthostatic tolerance through increased low‐frequency oscillations in mean arterial pressure and cerebral blood velocity (Lucaset et al., 2013; Russoet et al., 2017).

Despite these advances, critical gaps remain in our understanding of the temporal dynamics of integrated physiological responses to acute CBV reduction, particularly within the initial minutes of exposure. Although previous studies have examined steady‐state responses to LBNP, the crucial initial phase of cardiovascular adjustment remains poorly characterized. Our focus on the first 2 min is particularly relevant because: (1) orthostatic hypotension measurements taken within 1 min of standing are most strongly associated with dizziness and adverse outcomes (Jurascheket et al., 2017); (2) compensatory mechanisms are most active during this period; and (3) understanding these rapid responses could inform interventions to prevent orthostatic intolerance.

To address these knowledge gaps, we aimed to characterize the temporal dynamics of respiratory, cardiovascular and cerebrovascular responses during the first 2 min of LBNP‐induced CBV reduction in healthy young males. Based on the integrated physiological responses described above, we hypothesize that: (1) CBV reduction will trigger immediate respiratory adjustments, with $P_{\mathrm{ETC}O_{2}}$ decreasing rapidly owing to altered pulmonary perfusion patterns and modified chemoreflex sensitivity; (2) cerebral blood velocity will initially decrease owing to reduced $P_{\mathrm{ETC}O_{2}}$ but will be partly preserved through compensatory cardiovascular adjustments, including increased heart rate and altered breathing patterns; and (3) the temporal pattern of these integrated responses will reveal distinct temporal patterns of physiological compensation, with the most rapid adjustments occurring within the first 30–60 s of CBV reduction. By elucidating these mechanisms, we aim to provide critical insights into the adaptive responses by the body to haemodynamic challenges. These findings could potentially inform the development of more effective strategies for managing orthostatic hypotension and related conditions, improving quality of life for affected individuals and reducing the risk of falls in vulnerable populations.

## 2 MATERIALS AND METHODS

### 2.1 Ethical approval

The study conformed to the standards set by the latest revision of the *Declaration of Helsinki*, except for registration in a database. The Human Subjects Committee of Osaka Sangyo University (No. 2023‐02) granted ethical approval for the study. Eleven non‐smoking male university students participated in the research. Each subject provided written informed consent. All participants were free of known cardiovascular or pulmonary disorders, had no history of head injuries and were not taking any prescribed medication. Females were excluded from the study because previous studies have shown that women have lower orthostatic tolerance than men and have different heart rate (HR) responses, baroreceptor reflex responses, cerebral blood flow regulation and responsiveness to hormones when CBV is reduced (Goswami et al., 2019). We acknowledge this exclusion as a significant limitation that reduces the generalizability of our findings, and future studies will prioritize the inclusion of female participants with appropriate menstrual cycle phase control and protocol modifications to address this critical gap in physiological research.

### 2.2 Participants

The study included 11 young healthy males (mean ± SD age, 21.0 ± 1.0 years; height, 171.8 ± 8.2 cm; and weight, 64.6 ± 10.0 kg). Before each experiment, participants were instructed to abstain from strenuous exercise for 24 h, maintain their usual diet but avoid food with a high salt content, and arrive at the laboratory ≥2 h after a light meal.

### 2.3 Sample size calculation

A priori power analysis was conducted for primary physiological outcomes using G*Power 3.1.9.7 software. Based on previous LBNP studies examining cardiovascular responses to CBV reduction, we anticipated large effect sizes (Cohen's *d* ≥ 1.0) for primary cardiovascular variables (CBV, HR, blood pressure and $P_{\mathrm{ETC}O_{2}}$). For repeated‐measures ANOVA with α = 0.05, power = 80% and expected effect size *d* = 1.0, the required sample size was calculated to be *n* = 9. To account for potential dropouts and ensure robust statistical analysis, we recruited *n* = 11 participants. *Post hoc* power analysis confirmed adequate power (>85%) for all primary outcomes. The correlation analyses were conducted as exploratory investigations and were not included in the original power calculations.

### 2.4 Experimental protocol and procedure

The participants lay a supine position in the LBNP box, where the experiment was repeated three times, with a 15 min interval between the 3 min resting and 2 min LBNP conditions. This protocol was designed to confirm reproducibility within subjects and to improve measurement accuracy, given the relatively low sampling rate of respiratory data (∼12 breaths/min) in comparison to the 120 s analysis period. During the experiment, participants were instructed to avoid performing a Valsalva manoeuvre. In this study, a cylinder in the LBNP apparatus was used to enclose the lower half of the subject, who positioned himself in a supine position straddling a saddle placed within the cylinder. By reducing the pressure within the cylinder, a negative pressure was applied to the lower body. In LBNP conditions, unlike during upright positioning accompanied by postural changes, the soles of the feet did not touch the ground, in order to eliminate mechanical receptor stimulation from the lower‐limb muscles and input stimulation from the vestibular system. This experimental set‐up allows isolated evaluation of the effects of reduced central circulatory blood volume and observations of responses similar to those in an upright posture (Goswami et al., 2019). The design of the LBNP apparatus allows adjustment of suction levels, which can reduce cardiac preload by retaining venous blood in the lower body. During the LBNP conditions, the participant was encased from the xiphoid process downwards within the LBNP box, to which an LBNP of −45 mmHg was applied. This level of loading induces circulatory changes equivalent to orthostatic or moderately haemorrhagic states and is suitable for assessing distinct autonomical responses, such as heart rate and blood pressure regulation. In addition, −45 mmHg has been widely used to assess circulatory function after supine and space exposures (Feeley et al., 2024; Miyamoto et al., 2014; Ogoh et al., 2013). To ensure consistency and reproducibility with other studies, this method was selected. Throughout the experiment, changes in thoracic volume were estimated using impedance plethysmography, inclusive of LBNP. The application of pressure followed a standardized gradual reduction protocol targeting −45 mmHg over ∼30 s, followed by maintenance at target pressure. Analysis of pressure application kinetics revealed a progressive approach to the target: 68.0% ± 9.6% of target pressure achieved by 30 s, 90.7% ± 7.1% by 60 s, 94.2% ± 5.1% by 90 s, and 97.1% ± 4.7% by 120 s. Individual variability in pressure application decreased over time [coefficient of variation (CV): 14.2% ± 10.1% during 0–30 s, decreasing to 5.0% ± 4.0% during 90–120 s], indicating stabilization across participants. The reliability of LBNP application was assessed using intraclass correlation coefficients (ICC; 0.84–0.87) across three trials per participant (Table 1).

**TABLE 1:** Time course of reliability metrics for physiological parameters during lower‐body negative pressure.

Parameter		Time period of LBNP loading			
		0–30 s	30–60 s	60–90 s	90–120 s
LBNP load	ICC	0.85	0.86	0.84	0.87
	CV(%) ± SD	−14.2 ± 10.1	−8.3 ± 6.9	−5.5 ± 3.4	−5.0 ± 4.0
Systolic BP	ICC	0.90	0.89	0.88	0.89
	CV(%) ± SD	3.4 ± 2.1	5.5 ± 1.5	5.1 ± 2.4	6.1 ± 2.5
Diastolic BP	ICC	0.89	0.88	0.88	0.89
	CV(%) ± SD	5.6 ± 4.1	6.3 ± 3.0	6.3 ± 3.0	4.8 ± 2.4
Mean BP	ICC	0.90	0.89	0.88	0.90
	CV(%) ± SD	3.8 ± 2.6	5.0 ± 1.9	5.5 ± 2.5	4.6 ± 2.2
HR	ICC	0.92	0.91	0.9	0.91
	CV(%) ± SD	4.9 ± 2.6	4.5 ± 2.2	6.7 ± 2.3	5.2 ± 2.7
MCA *V*_mean_	ICC	0.89	0.88	0.87	0.88
	CV(%) ± SD	7.3 ± 3.9	10.0 ± 4.9	11.2 ± 4.9	11.1 ± 5.9
${\dot{V}}_{E}$	ICC	0.87	0.85	0.86	0.88
	CV(%) ± SD	21.7 ± 13.9	17.6 ± 13.8	19.7 ± 12.4	13.7 ± 8.0
*V*_T_	ICC	0.85	0.86	0.84	0.87
	CV(%) ± SD	22.4 ± 10.4	18.5 ± 7.9	23.6 ± 13.3	14.7 ± 7.6
RR	ICC	0.88	0.87	0.86	0.89
	CV(%) ± SD	15.3 ± 8.5	16.4 ± 8.1	14.0 ± 12.5	10.4 ± 7.9
$P_{\mathrm{ETC}O_{2}}$	ICC	0.89	0.87	0.88	0.91
	CV(%) ± SD	6.0 ± 3.6	5.8 ± 4.3	6.8 ± 3.6	3.9 ± 3.6

*Note*: Reliability criteria: Excellent (ICC > 0.90); Good (0.75 ≤ ICC ≤ 0.90). The LBNP values represent measurements from 11 subjects with three trials each. Abbreviations: CV, coefficient of variation; diastolic BP, diastolic blood pressure; HR, heart rate; ICC, intraclass correlation coefficient; LBNP, Lower‐body negative pressure load; MBP, mean blood pressure; MCA *V*_mean_, middle cerebral artery mean blood velocity; $P_{\mathrm{ETC}O_{2}}$_,_ end‐tidal $P_{CO_{2}}$; RR, respiratory rate; systolic BP, systolic blood pressure; ${\dot{V}}_{E}$_,_ minute ventilation; *V*_T_, tidal volume.

### 2.5 Experimental measurements

#### 2.5.1 Respiratory variables

All tests were conducted at constant room temperature between 23°C and 24°C, minimizing external stimuli. An automatic breath‐by‐breath gas analysing system (ARCO2000‐MET; Arcosystem, Chiba, Japan) recorded respiratory and metabolic data. This system consists of a differential pressure transducer, sampling tube, filter, suction pump and mass spectrometer. We digitized expired flow, CO_2_ and O_2_ concentrations at 200 Hz. Flow signals were converted to single‐breath data by matching to gas concentrations, accounting for the time lag (350 ms) in gas concentration measurements. From these measurements, we derived tidal volume (*V*_T_), respiratory rate (RR), ${\dot{V}}_{E}$ and $P_{\mathrm{ETC}O_{2}}$. Oxygen and CO_2_ measurements were calibrated using standard gas of known concentration before each test.

#### 2.5.2 Haemodynamic variables

The HR was monitored using a lead II ECG and measured by a cardiotachometer (AT601G; Nihon Kohden, Tokyo, Japan) triggered by the R wave on the ECG. All signals were recorded continuously using a personal computer in online mode at a sampling rate of 200 Hz during each test.  An automatic indirect manometer (EBP‐330, Minato Medical Science Co., Osaka, Japan) worn on the participant's left arm continuously recorded blood pressure (BP) every 30 s. Mean BP was calculated using the formula: (systolic BP − diastolic BP)/3 + diastolic BP. The auscultatory method using high‐precision microphone detection of Korotkoff sounds was selected to ensure measurement accuracy and minimize calibration errors that can occur with continuous monitoring during orthostatic stress. The MCA *V*_mean_ was monitored continuously using transcranial Doppler ultrasonography (WAKI, Atys Medical, St. Genislaval, France). A 2 MHz Doppler probe was positioned over the left temporal window and fixed with an adjustable headband and adhesive ultrasonic gel. Thoracic impedance was measured with a monitor (COCS‐31, Biotecs Co., Kyoto, Japan) at 10 µA and 20 kHz. Thoracic admittance (1/thoracic electrical impedance) served as an index of CBV (Cai et al., 2000; Miles & Gotshall, 1989).

### 2.6 Data analysis

All physiological data were collected initially at 200 Hz. Breath‐by‐breath respiratory measurements and beat‐by‐beat cardiovascular recordings were converted to 1 s intervals using linear interpolation between consecutive sampling points. For each participant, the interpolated data from three trials were time aligned to LBNP onset and averaged to create a single time series per variable. These individual time series from all 11 participants were then used for statistical analysis. The cardiorespiratory responses during LBNP were analysed in consecutive 30 s epochs. Baseline values were calculated by averaging the data for the first 3 min of resting conditions. Delta values for correlation analyses were calculated as (LBNP value − baseline value) for each time epoch. For cross‐correlation analysis, data were collected every 1 s for 2 min following LBNP onset and analysed using 10 s sliding windows with time lags ranging from −60 to +60 s. All data processing and analyses were performed offline using custom‐developed software.

### 2.7 Statistical analysis

#### 2.7.1 Primary analyses (a priori)

Primary analyses focused on temporal changes in cardiovascular and respiratory variables during LBNP exposure and were planned a priori based on our sample size calculations. We used one‐way repeated‐measures ANOVA to analyse changes in response variables over time during LBNP conditions. When a significant main effect was detected, post hoc comparisons were performed using Tukey's honestly significant difference (HSD) test to identify specific differences between baseline and each time point during LBNP. To control for multiple comparisons across primary outcomes, the false discovery rate (FDR) was controlled using the Benjamini–Hochberg procedure with α = 0.05. A total of 40 comparisons (10 parameters × 4 time points) were included in the FDR correction.

#### 2.7.2 Reliability assessment

The reliability of physiological measurements across the three trials was assessed using ICCs and CVs. The ICC was calculated using a two‐way mixed‐effects model for absolute agreement of single measurements. The CV was calculated as the SD divided by the mean and expressed as a percentage for each variable across the three trials. These reliability measures were computed for each 30 s epoch during LBNP exposure.

#### 2.7.3 Exploratory analyses (*post hoc*)

Exploratory correlation analyses were conducted *post hoc* to examine potential relationships between physiological variables and were not included in the original power calculations. Pearson's product–moment correlation coefficient (*r*) analysis was used to investigate the correlation between blood pressure changes and respiratory variables, and between respiratory parameters and cerebral blood flow. For mean BP–respiratory variable correlations, 12 comparisons were performed (3 variables × 4 time periods) with Benjamini–Hochberg FDR correction applied. Cross‐correlation analysis between respiratory parameters and MCA flow velocity was conducted using time lags ranging from −60 to +60 s, with significance determined using 95% confidence intervals (CIs; ±0.17) based on the assumption that one series represents white noise.

#### 2.7.4 Statistical software and reporting

Delta values used in the correlation and cross‐correlation analyses were calculated as (LBNP value − Baseline value). Data are presented as the mean ± SD. Statistical significance was set at an α‐level of 0.05 (5%). Significant differences between baseline and each time point after FDR correction are indicated in figures and tables. For correlation analyses, 95% CIs were calculated using Fisher's *z*‐transformation, and effect sizes were interpreted according to Cohen's conventions: small (|*r*| = 0.1–0.3), medium (|*r*| = 0.3–0.5) and large (|*r*| ≥ 0.5). All statistical analyses were performed using SPSS v.27.0 (IBM Corp.).

## 3 RESULTS

Table 1 shows the reliability of physiological measurements during LBNP, assessed using ICCs and CVs across three trials. All parameters showed good reliability across the four time periods (0–30, 30–60, 60–90 and 90–120 s) of LBNP exposure. Among respiratory parameters, $P_{\mathrm{ETC}O_{2}}$ demonstrated good reliability (ICC = 0.87–0.91) with relatively low variability (CV = 3.9%–6.8%). The ${\dot{V}}_{E}$ maintained good reliability (ICC = 0.85–0.88) despite higher variability (CV = 13.7%–21.7%). The *V*_T_ and RR also showed good reliability (*V*_T_: ICC = 0.84–0.87, CV = 14.7%–23.6%; RR: ICC = 0.86–0.89, CV = 10.4%–16.4%). For cerebrocardiovascular parameters, HR exhibited the highest reliability (ICC = 0.90–0.92, CV = 4.5%–6.7%), followed by mean arterial pressure (ICC = 0.88–0.90, CV = 3.8%–5.5%). Both systolic and diastolic BP maintained good reliability throughout (ICC = 0.88–0.90). The MCA *V*_mean_ measurements showed consistent reliability (ICC = 0.87–0.89) despite increasing CV values in later phases (7.3%–11.2%).

Figure 1 shows the temporal changes in physiological variables for a representative subject during an LBNP trial, and Figure 2 displays the percentage changes from baseline for these physiological variables (Table 2 provides detailed statistical comparisons). The CBV, as indicated by thoracic admittance, showed a rapid and significant decrease. Following FDR correction for multiple comparisons, thoracic admittance exhibited a sharp drop within the first 30 s, reaching ∼13.9% below baseline by the 30–60 s phase. This decrease then stabilized for the remainder of the LBNP period. In the final 90–120 s phase, thoracic admittance was reduced by 13.4% ± 3.6% from its baseline value, decreasing from 28.1 ± 9.4 × 10^−3^ to 24.5 ± 8.7 × 10^−3^ s. This sustained reduction indicates a significant decrease in CBV throughout the LBNP loading (*p* < 0.001, FDR‐corrected). Blood pressure responses were varied in the representative subject, with mean BP temporarily decreasing in the initial 30 s time period, systolic BP showing a slight decrease, and diastolic BP gradually increasing. Cardiovascular variables exhibited complex temporal responses in the group data. Mean BP showed a significant initial decrease (0–30 s: 92.2 ± 7.3 vs. 98.5 ± 7.6 mmHg, *p* < 0.01, FDR‐corrected), followed by rapid recovery to baseline levels and slight elevation by the final phase (overall change: +0.9 ± 6.2%). Systolic BP showed a non‐significant trend towards decrease during most phases, with significant reduction only at the final time point (−3.7% ± 5.5%, *p* = 0.028, FDR‐corrected). Diastolic BP decreased significantly during the initial phase (0–30 s: 77.5 ± 6.4 vs. 85.2 ± 7.0 mmHg, *p* < 0.01, FDR‐corrected), then gradually increased above baseline in later phases (final change: +4.4% ± 7.3%, *p* < 0.001, FDR‐corrected). The HR demonstrated a rapid initial increase in the representative subject, followed by a stepwise elevation throughout the LBNP period. The HR increased progressively and significantly throughout all phases after FDR correction, rising from 60.7 ± 9.5 beats/min at baseline to 70.4 ± 10.9 beats/min by 90–120 s, a 16.4% ± 10.6% increase (*p* < 0.001, FDR‐corrected). The MCA *V*_mean_ showed a slight decrease in the representative subject, suggesting a reduction in cerebral perfusion. The MCA *V*_mean_ showed no significant changes at any time point after FDR correction (−5.0% ± 8.4%, *p* = 0.372) over the course of LBNP exposure. Respiratory flow exhibited a suppressive trend in the individual subject, with a notable decrease in RR occurring immediately after initiation. After FDR correction, ${\dot{V}}_{E}$ showed overall temporal changes (*p* = 0.041), but no individual time points reached significance after FDR correction. The *V*_T_ remained stable throughout LBNP exposure, with no significant changes after FDR correction. The RR showed significant decreases during the initial phases after FDR correction, ending 5.7% ± 8.1% below baseline (*p* < 0.001, FDR‐corrected for early phases only). The representative subject showed fluctuations in $P_{\mathrm{ETC}O_{2}}$ levels, with a noticeable decrease from the baseline after the LBNP onset. The $P_{\mathrm{ETC}O_{2}}$ decreased consistently and significantly at all time points after FDR correction, from 38.2 ± 2.0 mmHg at baseline to 35.9 ± 2.1 mmHg at 90–120 s, a 5.9% ± 3.8% reduction (*p* < 0.001, FDR‐corrected).

**FIGURE 1:**
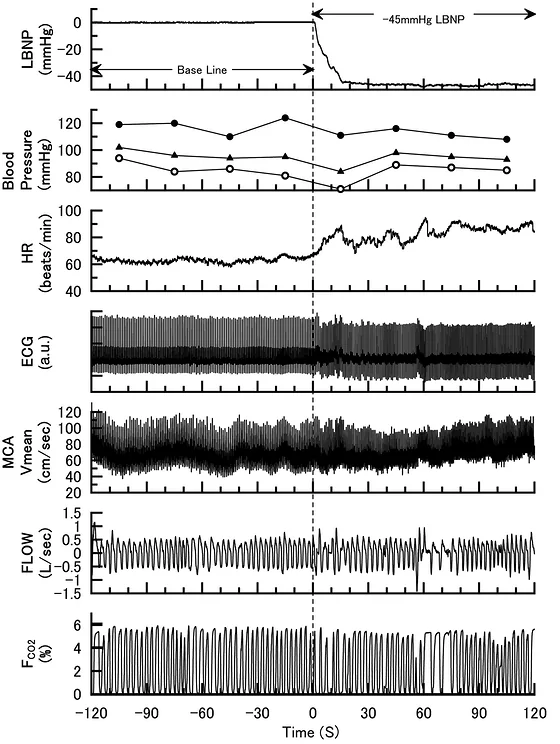
Temporal changes in physiological variables during one of three trials in a representative subject. This figure presents data from a single trial, illustrating the temporal changes in various physiological variables from 2 min before the intervention to 2 min after its onset. The variables displayed are (from top to bottom): LBNP, lower‐body negative pressure; blood pressure (filled circles, systolic; open circles, diastolic; filled triangles, mean; the blood pressure values are averaged over 30 s); flow; HR, heart rate; ECG; MCA *V*_mean_, middle cerebral artery blood flow velocity; FLOW, respiratory flow; and *F*_CO2_, fraction of inspired CO_2_. The *x*‐axis represents time, with zero marking the onset of LBNP, clearly distinguishing the baseline period from the LBNP application period.

**FIGURE 2:**
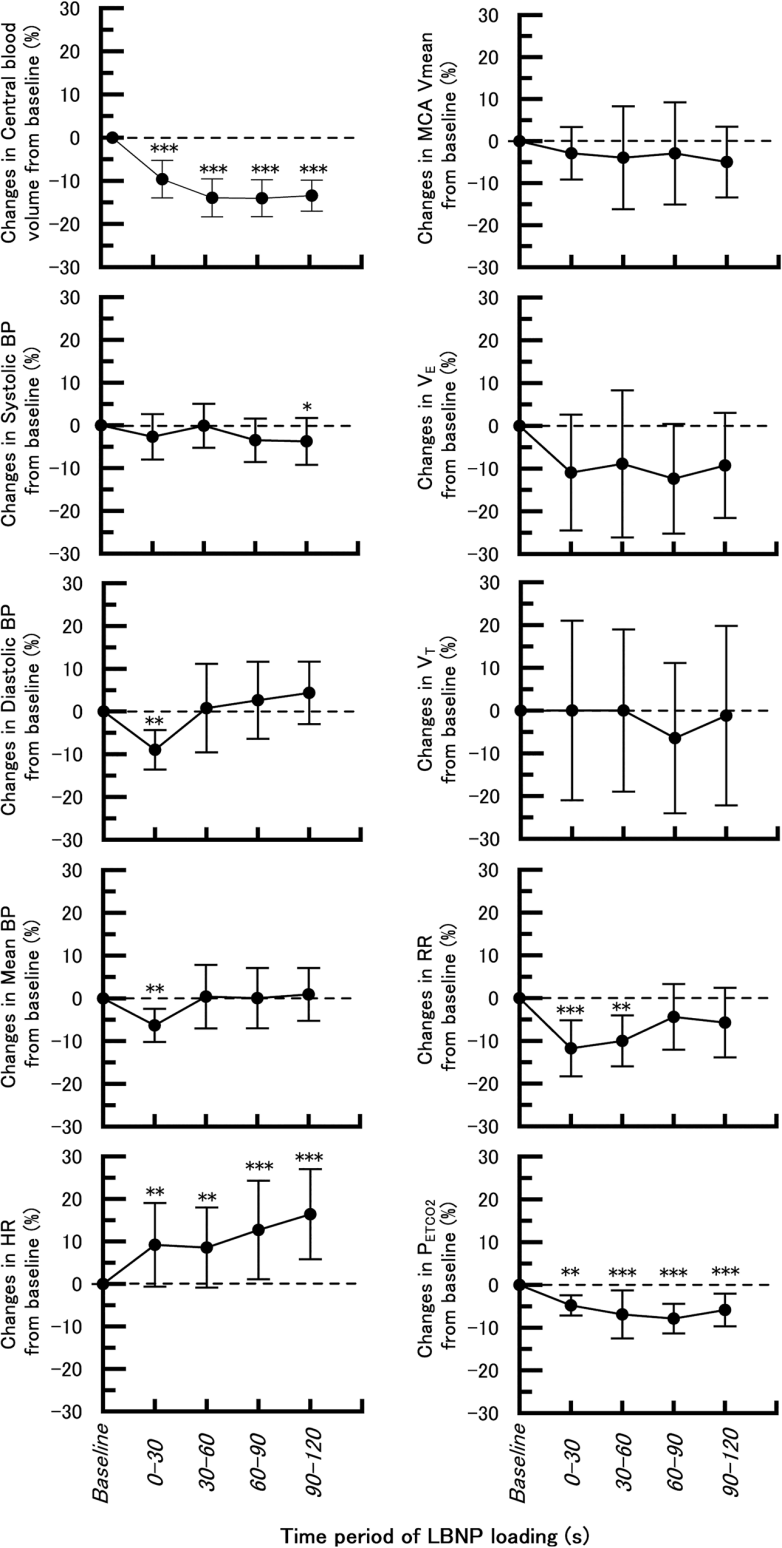
Time courses of cerebrocardiovascular and respiratory variables during baseline and lower‐body negative pressure (LBNP) periods. The graphs display percentage changes from baseline for the following variables: central blood volume; systolic BP, systolic blood pressure; diastolic BP, diastolic blood pressure; mean BP, mean blood pressure; HR, heart rate; MCA *V*_mean_, middle cerebral artery mean blood velocity; ${\dot{V}}_{E}$, minute ventilation; *V*_T_, tidal volume; RR, respiratory rate; and $P_{\mathrm{ETC}O_{2}}$, end‐tidal $P_{CO_{2}}$. Data points represent mean values averaged over 30 s intervals: baseline, 0–30 s, 30–60 s, 60–90 s and 90–120 s of LBNP application. Error bars indicate the SD. The horizontal line at 0% on the *y*‐axis represents baseline values. Statistical significance was assessed using one‐way repeated‐measures ANOVA followed by Tukey's *post hoc* tests comparing each time point to baseline. Multiple comparison correction was applied using the Benjamini–Hochberg false discovery rate (FDR) method. Symbols indicate significance after FDR correction: ^*^ *p* < 0.05, ^**^ *p* < 0.01 and ^***^ *p* < 0.001. Total number of comparisons for FDR correction: 10 parameters × 4 time points = 40 comparisons. Non‐significant changes (*p* > 0.05 after FDR correction) are shown without statistical symbols. The FDR correction controls the expected proportion of false discoveries among all rejected hypotheses at α = 0.05.

**TABLE 2:** Comparison of respiratory, metabolic responses, cardiovascular and cerebral blood flow responses during Baseline and lower‐body negative pressure.

Parameter				Time period of LBNP loading															One‐way repeated ANOVA
	Baseline			0–30 s				30–60 s				60–90 s				90–120 s			(*P*‐value)
LBNP load, mmHg		0		−30.6	±	4.1^***^		−40.8	±	3.2^***^		−42.4	±	2.3^***^		−43.7	±	2.1^***^	< 0.001
				(68% target)				(91% target)				(94% target)				(97% target)			
Systolic BP, mmHg	125	±	10	122	±	11.2		125	±	12.3		121	±	12.6		120	±	13.0^*^	0.028
Diastolic BP, mmHg	85.2	±	7	77.5	±	6.4^**^		85.6	±	8.9		87.3	±	9		88.8	±	8	< 0.001
Mean BP, mmHg	98.5	±	7.6	92.2	±	7.3^**^		98.7	±	9		98.4	±	9.6		99.3	±	9.4	0.001
HR, beats/min	60.7	±	9.5	66.1	±	10.9^**^		65.9	±	11.6^**^		68.2	±	11.3^***^		70.4	±	10.9^***^	< 0.001
MCA *V*_mean_, cm/s	56.4	±	15.9	54.6	±	15.4		54.1	±	16.5		54.9	±	17.2		53.6	±	15.9	0.372
${\dot{V}}_{E}$, L/min	9.7	±	1.6	8.7	±	1.9		8.8	±	2		8.4	±	1.3		8.9	±	1	0.041
*V*_T_, mL	570	±	93.6	578	±	193		567	±	132		538	±	168		566	±	175	0.835
RR, breaths/min	18.1	±	3.1	15.8	±	2.2^***^		16.3	±	3.2^**^		17.3	±	3.3		17.1	±	3.7	< 0.001
$P_{\mathrm{ETC}O_{2}}$, mmHg	38.2	±	2	36.3	±	1.8^**^		35.5	±	2.5^***^		35.1	±	2.1^***^		35.9	±	2.1^***^	< 0.001

*Note*: Values are means ± SD. Data were analysed using repeated‐measures ANOVA followed by Tukey's *post hoc* test. Multiple comparison correction was applied using the Benjamini–Hochberg false discovery rate method (40 total comparisons across all measured variables). Target LBNP was −45 mmHg; values in parentheses show percentage of target pressure achieved during each time period. Abbreviations: BP, blood pressure; HR, heart rate; LBNP, supine lower‐body negative pressure; MCA *V*_mean_, middle cerebral artery mean blood velocity; $P_{\mathrm{ETC}O_{2}}$, end‐tidal $P_{CO_{2}}$; RR, respiratory rate; ${\dot{V}}_{E}$, minute ventilation; *V*_T_, tidal volume.

*p < 0.05, **p < 0.01, ***p < 0.001, significant difference from baseline after false discovery rate correction.

Figure 3. Temporal relationships between changes in mean blood pressure and respiratory variables during lower body negative pressure (LBNP). Scatter plots show correlations between changes in mean BP (*x*‐axis) and respiratory variables (*y*‐axis) for each 30‐second period. Variables are expressed as delta values from baseline. Pearson correlation analysis with Benjamini‐Hochberg FDR correction was applied (12 comparisons: 3 variables × 4 time periods). Significant correlations after FDR correction are marked with asterisks and shown with regression lines: *V*_T_ at 0–30s (*r* = −0.78, 95% CI: −0.92 to −0.47, *p* = 0.004, large effect). Non‐significant correlations after FDR correction are shown without regression lines. Dotted lines indicate zero reference. *n* = 11 for all analyses.

**FIGURE 3:**
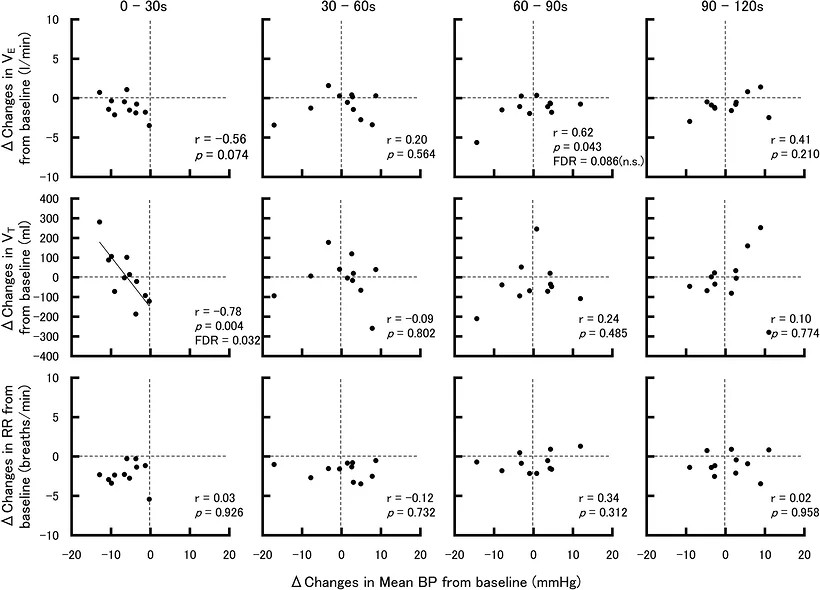
Temporal relationships between changes in mean blood pressure and respiratory variables during lower‐body negative pressure (LBNP). Scatter plots show correlations between changes in mean blood pressure (BP) (*x*‐axis) and respiratory variables (*y*‐axis) for each 30 s period. Variables are expressed as delta values from baseline. Pearson correlation analysis with Benjamini–Hochberg false discovery rate (FDR) correction was applied (12 comparisons: 3 variables × 4 time periods). Significant correlations after FDR correction are marked with asterisks and shown with regression lines: *V*_T_ at 0–30 s (*r* = −0.78, 95% CI: −0.92 to −0.47, *p* = 0.004). Non‐significant correlations after FDR correction are shown without regression lines. Dotted lines indicate zero reference. *n* = 11 for all analyses.

Figure 4. Cross‐correlation analysis between MCA V_mean_ and respiratory parameters during lower body negative pressure. Cross‐correlation coefficients are shown for (A) MCA V_mean_ and minute ventilation (VE), (B) MCA V_mean_ and tidal volume (*V*_T_), and (C) MCA V_mean_ and respiratory rate (RR). Data were collected every second for 2 minutes following LBNP onset (time 0) and averaged over 10‐second intervals for analysis. Dotted horizontal lines represent 95% confidence intervals (±0.17). Negative time lags indicate that respiratory changes precede MCA V_mean_ changes. Significant negative correlations (*p* < 0.05) are observed for *V*_E_ and *V*_T_ at time lags of 10–20 seconds, indicating that respiratory changes temporally precede cerebrovascular responses. This temporal pattern suggests respiratory‐mediated modulation of cerebrovascular function during circulatory stress.

**FIGURE 4:**
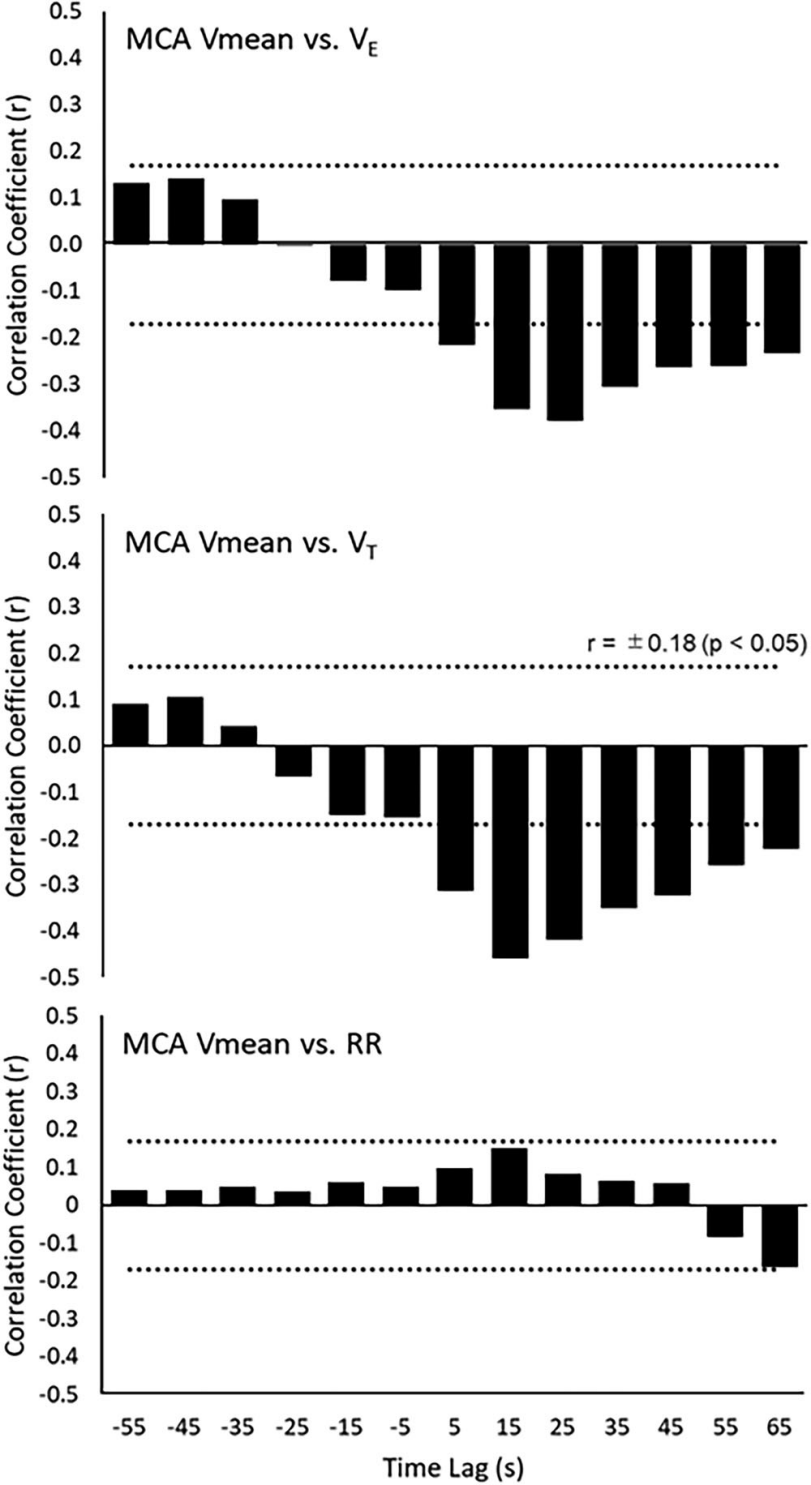
Cross‐correlation analysis between middle cerebral artery mean blood velocity (MCA *V*_mean_) and respiratory parameters during lower‐body negative pressure. Cross‐correlation coefficients are shown for MCA *V*_mean_ and minute ventilation (${\dot{V}}_{E}$), tidal volume (*V*_T_) and respiratory rate (RR). Data were collected 1 s for 2 min following lower‐body negative pressure onset (time 0) and averaged over 10 s intervals for analysis. Dotted horizontal lines represent 95% confidence intervals (±0.17). Negative time lags indicate that respiratory changes precede MCA *V*_mean_ changes. Significant negative correlations (*p* < 0.05) are observed for ${\dot{V}}_{E}$ and *V*_T_ at time lags of 10–20 s, indicating that respiratory changes temporally precede cerebrovascular responses. This temporal pattern suggests respiratory‐mediated modulation of cerebrovascular function during circulatory stress.

## 4 DISCUSSION

This study provides a detailed analysis of the temporal dynamics of respiratory, cardiovascular and cerebrovascular responses during the initial 2 min following the onset of LBNP. Key findings reveal novel compensatory mechanisms: (1) a strong negative correlation between mean blood pressure and tidal volume (*r* = −0.78, *p* = 0.004) during the initial 30 s; and (2) temporal patterns suggesting that respiratory changes precede cerebrovascular responses by 10–20 s, as revealed by cross‐correlation analysis. Our findings offer insights into the intricate interplay between these physiological systems in response to acute CBV reduction, with a particular emphasis on respiratory dynamics and the identification of distinct temporal patterns of physiological compensation.

### 4.1 Temporal mechanisms of cardiorespiratory–cerebrovascular integration

Our findings reveal a temporal cascade of physiological responses to LBNP‐induced CBV reduction. The initial haemodynamic changes, characterized by decreased mean BP (0–30 s: *p* = 0.005, FDR‐corrected) following LBNP onset, are likely to have triggered arterial baroreceptor unloading. Previous animal studies have demonstrated that carotid sinus baroreceptor stimulation can directly modulate respiratory control through connections with medullary respiratory neurons (Brunner et al., 1982). This baroreceptor–respiratory coupling (referring to the interaction whereby changes in blood pressure influence breathing patterns via central neural pathways) might contribute to the rapid respiratory adjustments observed during the initial period of LBNP exposure. Subsequently, complex changes in pulmonary circulation and CO_2_ dynamics emerged. The sustained reduction in CBV (13.4% throughout LBNP) is likely to have altered pulmonary ventilation–perfusion relationships, leading to enhanced CO_2_ washout from the lungs. Our previous work (Miyamoto et al., 2014) demonstrated that CBV reduction shifts the respiratory plant element, indicating changes in the efficiency of pulmonary CO_2_ removal. The significant negative correlation between mean BP and *V*_T_ during the initial phase (*r* = −0.78, *p* = 0.004, FDR‐corrected) suggests that decreased cardiac output and altered pulmonary perfusion might have enhanced the efficiency of CO_2_ elimination per unit of ventilation, contributing to the observed $P_{\mathrm{ETC}O_{2}}$ reduction despite stable ${\dot{V}}_{E}$. Cerebrovascular responses developed over a longer time course. The reduced $P_{\mathrm{ETC}O_{2}}$ (5.9% decrease, *p* < 0.001, FDR‐corrected) is likely to have contributed to cerebral vasoconstriction through CO_2_‐mediated mechanisms. Although MCA *V*_mean_ showed only a non‐significant trend towards a decrease (−5.0%, *p* = 0.372), the cross‐correlation analysis revealed that respiratory changes preceded cerebrovascular adjustments by 10–20 s. This temporal pattern suggests that $P_{\mathrm{ETC}O_{2}}$‐mediated cerebral vasoconstriction might have offset, in part, the cerebral hypoperfusion that would otherwise result from reduced cardiac output, representing an adaptive mechanism to maintain cerebral perfusion pressure during central hypovolaemia.

### 4.2 Changes in CBV and haemodynamic responses

The observed decrease in CBV attributable to LBNP loading in healthy individuals was ∼13.4%. This result is consistent with the acute time course of our study, albeit lower than findings from previous studies, in which non‐athletes showed decreases ranging from 18.3% to 24.5% (Feeley et al., 2024; Miyamoto et al., 2014). The difference in the rate of CBV shift between studies, despite identical LBNP loading conditions (−45 mmHg), can be attributed to the difference in LBNP loading time (2 min in the present study vs. 10 min in previous studies). A non‐significant decrease in MCA *V*_mean_ was observed after FDR correction, with MCA *V*_mean_ decreasing by 2.8 cm/s (−5.0%), from 56.4 to 53.6 cm/s. These results fall within the range of cerebral blood flow decrease rate reported by Lucas et al. (2013) in their orthostatic tolerance test. Mean BP decreased temporarily from 98.5 ± 7.6 to 92.2 ± 7.3 mmHg (−6.3%) during the 0–30 s phase, consistent with previous studies reporting that mean BP begins to decrease significantly at 40% of maximum LBNP (McManus et al., 2008), and that even mild LBNP loading of −20 mmHg can temporarily lower mean BP and activate the arterial baroreceptor reflex (Hisdal et al., 2002).

### 4.3 Respiratory dynamics and their significance

Our study revealed comprehensive dynamic responses of respiratory regulation to LBNP loading. After rigorous FDR correction for multiple comparisons, respiratory variables showed distinct temporal patterns, with immediate decreases in the early phase (0–30 s) particularly in RR (significant after FDR correction), followed by non‐significant changes in ${\dot{V}}_{E}$, and a consistent reduction in $P_{\mathrm{ETC}O_{2}}$ (−5.9%) throughout the exposure period (significant at all time points after FDR correction). These statistically robust changes in respiratory variables highlight the crucial role of the respiratory system in compensating for acute haemodynamic challenges, an aspect that has been largely overlooked in previous studies that have focused primarily on cardiovascular adaptations.

Notably, we found a significant negative correlation between mean BP and *V*_T_ exclusively during the initial phase of LBNP (*r* = −0.78, *p* = 0.004, FDR‐corrected), which disappeared in subsequent periods. These phase‐specific correlations during the initial 30 s suggest a coordinated cardiovascular–respiratory response to acute orthostatic stress. This temporal specificity of the respiratory response, particularly the association between blood pressure changes and *V*_T_ during the acute phase, suggests the existence of rapid respiratory compensation mechanisms during orthostatic stress.

### 4.4 Integrated mechanisms and controller–plant interactions

Building on our previous respiratory equilibrium analysis (Miyamoto et al., 2014), the present findings suggest a complex interaction between controller and plant elements during acute CBV reduction. The leftward shift of the respiratory operating point during LBNP appears to result from: (1) direct baroreceptor‐mediated modification of the respiratory controller; (2) altered pulmonary ventilation–perfusion relationships enhancing CO_2_ elimination efficiency (plant element modification); and (3) reduced cerebral blood flow attenuating central chemoreceptor CO_2_ washout, which, paradoxically, might enhance chemosensitivity to maintain respiratory drive despite reduced $P_{\mathrm{ETC}O_{2}}$. This integrated cardiorespiratory response pattern might represent an evolutionary adaptation to maintain cerebral perfusion during hypovolaemic stress. The rapid reduction in $P_{\mathrm{ETC}O_{2}}$ through enhanced pulmonary CO_2_ washout provides cerebral vasoconstriction that helps to preserve cerebral perfusion pressure when systemic blood pressure falls. However, this mechanism operates within narrow physiological limits, because excessive hypocapnia could impair cerebral autoregulation and compromise cognitive function.

### 4.5 Compensatory mechanisms and physiological roles

Following the observed 13.4% decrease in CBV during LBNP exposure, our analysis revealed a sequential activation of compensatory mechanisms. During LBNP exposure, we observed significant decreases in multiple respiratory variables: ${\dot{V}}_{E}$ decreased by 0.8 L/min (−9.3%), from 9.7 to 8.9 L/min in the final phase (90–120 s); $P_{\mathrm{ETC}O_{2}}$ showed a consistent reduction of 2.3 mmHg (5.9%), from 38.2 ± 2.0 to 35.9 ± 2.1 mmHg; and RR demonstrated an initial decrease of 11.7% during the early phase (0–30 s). These rapid and significant changes highlight the crucial role of the respiratory system in compensating for acute haemodynamic challenges, an aspect that has been largely overlooked in previous studies. The temporal pattern of these respiratory changes suggests a biphasic response to orthostatic stress. The initial phase is characterized by immediate decreases in respiratory variables, followed by partial recovery, particularly in RR. This pattern is likely to reflect an immediate reflex response, followed by the engagement of more complex, integrated compensatory mechanisms involving baroreflex and chemoreflex pathways.

Our findings demonstrate multiple integrated compensatory mechanisms during LBNP‐induced central hypovolaemia. We observed a unique temporal pattern in the relationship between blood pressure and respiratory responses. We observed a significant negative correlation between mean BP and *V*_T_ during the initial phase of LBNP (*r* = −0.78, *p* = 0.004). Although correlation does not imply causation, this temporal association during the initial phase, along with individual variability in responses, might reflect the presence of physiological interactions between blood pressure regulation and respiratory control. This relationship was specific to the initial phase (0–30 s) and disappeared in subsequent periods, suggesting that some individuals might exhibit coordinated cardiovascular and respiratory responses during the acute phase of orthostatic stress. This correlation, which was present only in the first 30 s and disappeared in subsequent periods, indicates an acute respiratory adjustment mechanism specific to the initial phase of orthostatic stress.

These respiratory responses appear to serve two primary physiological roles. First, the modulation of breathing patterns, particularly through changes in *V*_T_, might enhance venous return through the respiratory pump mechanism. Second, the observed decrease in $P_{\mathrm{ETC}O_{2}}$ suggests that CO_2_‐mediated vasoconstriction acts as an independent regulatory mechanism parallel to pressure‐dependent cerebral autoregulation during the initial phase of LBNP. This moderate decrease in $P_{\mathrm{ETC}O_{2}}$ (from 38.2 ± 2.0 to 35.9 ± 2.1 mmHg) appears to be physiologically significant for two reasons: (1) it operates within the optimal range for cerebral blood flow regulation between normocapnic ($P_{CO_{2}}$ = 37.1 mmHg) and mildly hypocapnic conditions; and (2) this magnitude of change is sufficient to induce cerebral vasoconstriction while avoiding the potential adverse effects of excessive hypocapnia ($P_{CO_{2}}$ = 22.2 mmHg) on autoregulation (Aaslid et al., 1989).

Our identification of this temporal transition in compensatory mechanisms, from initial cardiovascular responses to subsequent respiratory‐mediated regulation, extends previous findings on cerebral hypoperfusion and respiratory control. These findings are supported by our previous work examining the effects of cerebral hypoperfusion on the respiratory chemoreflex during orthostatic stress (Feeley et al., 2024; Miyamoto et al., 2014). The leftward shift of the ${\dot{V}}_{E}$–$P_{\mathrm{ETC}O_{2}}$ relationship during LBNP, without changes in chemoreflex sensitivity, indicates an enhanced ventilatory response for any given $P_{\mathrm{ETC}O_{2}}$ level during orthostatic stress. This observation is particularly relevant given that our present study showed a non‐significant decrease in MCA *V*_mean_ by 5.0% ± 8.4% during LBNP exposure, suggesting a link between an index of cerebral perfusion and respiratory control. It is also supported by the observation that increased *V*_T_ uses a so‐called ‘respiratory pump’, resulting in a greater decrease in intrathoracic pressure (i.e., greater vacuum), increases blood flow in the inferior vena cava, improves venous return and is associated with increased resistance to LBNP (Moreno et al., 1967; Rickards et al., 2008). Although the precise mechanisms underlying these respiratory responses to acute LBNP loading remain largely unclear in humans, animal studies have provided important insights. Specifically, studies in dogs have demonstrated that changes in carotid sinus pressure affect both RR and ${\dot{V}}_{E}$ (Brunner et al., 1982), suggesting that similar mechanisms might be operating in humans during orthostatic stress (Javorka et al., 2018). This mechanism provides a physiological basis for the respiratory responses we observed, highlighting the intricate interplay between cerebrovascular and respiratory regulation during orthostatic challenges.

### 4.6 Temporal cascade and cross‐correlation evidence

The analysis revealed a temporal cascade: LBNP‐induced haemodynamic changes → respiratory adjustment → cerebrovascular response (10–20 s delay), as demonstrated by cross‐correlation analysis between respiratory and cerebrovascular variables. This sequence supports the hypothesis that baroreceptor‐mediated respiratory modifications serve as an intermediate mechanism linking cardiovascular and cerebrovascular responses. Although the correlations observed should be interpreted cautiously given their exploratory nature, they provide preliminary evidence for rapid physiological coupling that warrants further investigation in larger, hypothesis‐driven studies.

### 4.7 Limitations and future directions

Several limitations should be considered when interpreting the results of this study.

#### 4.7.1 Statistical power and sample size considerations

Although a priori power calculations demonstrated adequate power (>85%) for primary outcomes, the exploratory correlation analyses were conducted *post hoc*, without formal power calculations. The study might have been underpowered to detect moderate correlation coefficients (*r* = 0.4–0.6) reliably, and the observed correlations should be interpreted as hypothesis‐generating rather than conclusive evidence. Future studies should use larger sample sizes (*n* ≥ 20–25) for correlation analyses.

#### 4.7.2 Participant characteristics and generalizability

All participants were healthy young males, limiting generalizability to women and older adults, who represent populations most affected by orthostatic hypotension. Although sex differences in orthostatic tolerance exist (Goswami et al., 2019), the exclusion of women was based on establishing clear baseline responses in a homogeneous population. Previous studies show that women have lower LBNP tolerance (58% completion rate at −45 mmHg; Russomano et al., 2015), reflecting physiological differences rather than safety limitations. Importantly, women exhibit distinct orthostatic response patterns, including different cardiorespiratory regulation strategies, in comparison to men. Female sex hormones significantly modulate these responses, with oestrogen promoting vasodilatation and menstrual cycle fluctuations creating temporal variability in orthostatic tolerance. Given these fundamental sex differences, our findings in young males cannot be extrapolated directly to women, and the phase‐specific compensatory mechanisms we identified might manifest differently in females. Future research should include both sexes, with appropriate protocol modifications and systematic assessment of menstrual cycle effects. We are committed to addressing this limitation in subsequent investigations by implementing sex‐balanced study designs that account for hormonal influences on cardiovascular and respiratory responses to orthostatic stress.

#### 4.7.3 Methodological considerations

Blood pressure measurements were taken at 30 s intervals using automated cuff‐based sphygmomanometry rather than continuous beat‐to‐beat monitoring. This methodological approach was selected for several reasons: (1) auscultatory measurements using Korotkoff sound detection provide highly accurate absolute blood pressure values, without calibration drift and motion artefacts commonly associated with continuous finger photoplethysmography during LBNP; (2) automated cuff measurements offer superior accuracy for detecting clinically relevant blood pressure changes during orthostatic stress; and (3) the 30 s interval was appropriate for capturing the primary haemodynamic responses while maintaining measurement stability across multiple trials. Although this approach might have limited detection of rapid haemodynamic fluctuations occurring within the first 10–20 s of LBNP onset, it provided stable measurements with high reliability (CV < 6.7%) that were well suited for analysis of cardiovascular responses to CBV reduction.

#### 4.7.4 LBNP protocol limitations

The 2 min exposure duration, although appropriate for examining acute responses, might have been insufficient to observe longer‐term adaptive mechanisms or steady‐state adjustments. Although application of LBNP showed some individual variability during the initial phase (CV = 14.2% at 0–30 s), this stabilized rapidly (CV = 5.0% by 90–120 s) and was accounted for through the repeated‐measures design and high measurement reliability (ICC > 0.84). Longer exposure durations might reveal additional adaptation patterns, and we believe that future studies should use multiple levels of LBNP to evaluate how varying degrees of CBV reduction affect respiratory and cerebrovascular responses.

#### 4.7.5 Analytical limitations

The temporal relationships observed in cross‐correlation analysis should be considered preliminary evidence, requiring confirmation in larger studies. The correlational nature of these analyses cannot establish definitive causality, and the exploratory nature increases the risk of type I error, requiring replication in confirmatory studies.

Future research should: (1) expand to include female participants and older adults, with systematic assessment of hormonal status; (2) incorporate continuous blood pressure monitoring; (3) extend LBNP exposure duration; (4) include direct measurements of proposed mechanisms (intrathoracic pressure, cardiac output and cerebral oxygenation); and (5) conduct comprehensive power analyses for optimal sample sizes.

Despite these limitations, our study design, with multiple trials per participant, controlled conditions and high measurement reproducibility, provides valuable insights into integrated cardiorespiratory–cerebrovascular responses during acute central hypovolaemia in healthy young males.

### 4.8 Clinical implications and future directions

These findings highlight the clinical importance of the first minute of orthostatic stress, aligning with recommendations for early assessment (Juraschek et al., 2017). Given that orthostatic hypotension affects nearly one in five community‐dwelling older adults (Saedon et al., 2020), the temporal compensatory mechanisms identified might inform targeted interventions for managing orthostatic intolerance in clinical populations (Wieling et al., 2022). The rapid respiratory adjustments observed represent potential therapeutic targets through respiratory training interventions.

## 5 CONCLUSION

In conclusion, our study provides the first comprehensive evidence for temporal cardiorespiratory–cerebrovascular integration during LBNP‐induced central hypovolaemia in healthy young males. The identification of distinct temporal patterns of compensation provides a new framework for understanding rapid physiological adaptations to orthostatic stress. The observed changes in CBV, blood pressure and respiratory variables after rigorous statistical correction demonstrate the rapid activation of compensatory mechanisms, highlighting the intricate interplay between respiratory control, cardiovascular adjustments and cerebrovascular regulation in maintaining homeostasis during acute haemodynamic challenges.

## AUTHOR CONTRIBUTIONS

M.F.: Conceptualization; formal analysis; investigation; writing—original draft; writing—review and editing. G.I.: Formal Analysis; investigation; supervision; writing—review and editing. A.S.: Formal Analysis; investigation; supervision; writing—review and editing. T.S.: Resources; investigation; supervision; funding acquisition; writing—review and editing. H.N.: Investigation; supervision; funding acquisition; writing—review and editing. S.O.: Investigation; supervision; funding acquisition; writing—review and editing. T.M.: Conceptualization; formal Analysis; investigation; funding acquisition; methodology; writing—original draft, Writing—review and editing. All approved the final version of the manuscript and agree to be accountable for all aspects of the work in ensuring that questions related to the accuracy or integrity of any part of the work are appropriately investigated and resolved. All persons designated as authors qualify for authorship, and all those who qualify for authorship are listed.

## CONFLICT OF INTEREST

None declared.

## Data Availability

The data that support the findings of this study are available from the corresponding author upon reasonable request. The data are not publicly available owing to privacy and ethical restrictions, because they contain information that could compromise research participant privacy.
